# Preparation and Characterization of Mg-Doped Calcium Phosphate-Coated Phycocyanin Nanoparticles for Improving the Thermal Stability of Phycocyanin

**DOI:** 10.3390/foods11040503

**Published:** 2022-02-10

**Authors:** Qian Li, Ping Dong, Laihao Li

**Affiliations:** 1 College of Food Science and Engineering, Ocean University of China, Qingdao 266003, China; liqianlq1990@163.com; 2 Key Laboratory of Aquatic Product Processing, Ministry of Agriculture and Rural Affairs, National R&D Center for Aquatic Product Processing, South China Sea Fisheries Research Institute, Chinese Academy of Fishery Sciences, Guangzhou 510300, China

**Keywords:** phycocyanin, calcium phosphate, in situ mineralization, Ca/P ratio, thermal stability

## Abstract

Phycocyanin (PC) is a blue-colored, pigment-protein complex with unique fluorescence characteristics. However, heat leads to PC fading and fluorescence decay, hampering its widespread application. To improve the thermal stability of PC, we induced the in situ mineralization of calcium phosphate (CaP) on the PC surface to prepare PC@Mg-CaP. The nanoparticles were characterized using transmission electron microscopy, energy dispersive spectrometry, fourier transform infrared spectroscopy, and X-ray diffraction. The results showed that PC@Mg-CaP was spherical, and the nanoparticle size was less than 200 nm. The shell of PC@Mg-CaP was composed of amorphous calcium phosphate (ACP). The study suggested that CaP mineralization significantly improved the thermal stability of PC. After heating at 70 °C for 30 min, the relative concentration of PC@Mg-CaP with a Ca/P ratio = 2 was 5.31 times higher than that of PC. Furthermore, the Ca/P ratio was a critical factor for the thermal stability of PC@Mg-CaP. With decreasing Ca/P, the particle size and thermal stability of PC@Mg-CaP significantly increased. This work could provide a feasible approach for the application of PC and other thermal-sensitive biomolecules in functional foods requiring heat treatment.

## 1. Introduction

Phycocyanin (PC) is a bright blue-colored, water-soluble, pigment-protein complex that is used as a natural pigment and fluorescent marker owing to its unique physical and chemical properties. In recent years, studies have shown that PC also has a variety of biological functions, including antioxidant [1,2], anti-inflammatory [3], antitumor [4], and liver protective activities [5]. These findings indicate that PC may have great potential applications in functional food, biological detection, and medicine fields. However, PC is highly sensitive to the environment, and its stability is significantly affected by temperature, pH, and ultraviolet radiation [2]. Processing-related heat generation is a universal phenomenon. Consequently, heat-lability becomes the primary factor hindering industrial applications of PC. Even at neutral pH, a temperature higher than 45 °C will cause PC denaturation, leading to the blue color fading [6]. Therefore, finding ways to enhance the thermal stability of PC has become an urgent issue.

In recent years, nanotechnology has been explored to enhance the stability of PC [7]. Microencapsulation could slow down the degradation of PC at high temperatures and increase the shelf life of the colorant. PC microencapsulated in sodium alginate (2.5%) was heated at 50 °C for 30 min, and the relative concentration (C_R_) of PC was found to be 94.74% [8]. In vitro studies showed that liposomes prepared with soybean phosphatidylcholine and cholesterol that contained 2% PC increased the accumulation of PC in the cuticle and throughout human skin by nearly 50% as compared with untreated PC [9]. However, the preparations of most reported PC carriers are complex and expensive. These require large-scale precision instruments, such as homogenizers and spray dryers, to achieve nuanced control of production [10,11]. Some nanocarriers also have associated potential stability problems such as lipid oxidation and drug leakage [12]. These also present great challenges for large-scale production. In addition, chloroform [9,13], ammonium molybdate [14], and other toxic reagents have been used in the preparation processes, which also raises issues concerning the biological safety of the nanocarriers. Hence, finding a safe, stable, and inexpensive approach to improve stability is the key to solving limitations associated with the development and applications of PC. 

Biomineralization is a ubiquitous phenomenon in nature [15,16]. The mineralized shell provides eggs, algae, and bacteria with special properties, such as heat resistance and mechanical protection, so that fragile organisms can tenaciously adapt and survive in adverse environments such as high temperatures [17,18]. Biomimetic mineralization can artificially provide mineral shells for organisms and materials with insufficient or nonexistent mineralization ability [19]. Compared with traditional nanotechnology, biomimetic mineralization only requires a biosafety mineral solution, and the production process is simple with a low cost [18]. Biomimetic mineralized hybrids also have high biocompatibility [20,21]. At present, biomimetic mineralization has been applied to vaccine improvement [22], cell protection [23], tissue repair [24], and drug delivery [25]. Notably, the mineralized shell could increase the thermal stability of inclusions by reducing the hydrogen bond exchange rate between the internal protein and water molecules in the environment. When heated at 65 °C for 30 min, the relative activity of mineralized catalase using calcium phosphate (CaP) was found to be nearly seven times higher than that of the untreated group [26]. The silicified EV71 virus vaccine could still maintain 90% efficacy after 35 days at room temperature, overcoming the restriction of vaccines needing to be stored at low temperatures [27]. Inspired by biomineralization, we expected to improve the thermal stability of PC with in situ mineralized CaP. CaP exhibits high biocompatibility and good biodegradability because it is the main inorganic constituent of bone and teeth. It can participate in normal metabolism in the living body by being dissolved into nontoxic ions [28]. Furthermore, CaP is easily and inexpensively produced, which makes it a highly attractive biomineralization nanocarrier [24].

The aim of this work was to characterize the structure of mineralized PC nanoparticles and to evaluate the effect of biomineralization on the thermal stability of PC. The underlying protective mechanism is also discussed considering the physical properties of the mineralized protein. This study provides a method for the application of PC in functional foods that require heat treatment such as pasteurization. In addition, this approach does not involve toxic reagents. The simple controllable production process and low cost make it suitable for industrial production. Based on these advantages, this strategy might have great potential for improving the heat-lability of biomolecules under thermal stress.

## 2. Materials and Methods

### 2.1. Materials and Chemicals

PC with purity (A_620_ nm/A_280_ nm) > 2.0, was purchased from Zhejiang Binmei Biotechnology Co., Ltd. (Zhejiang, China). Anhydrous calcium chloride (CaCl_2_), magnesium chloride hexahydrate (MgCl_2_·6H_2_O), and hydrochloric acid (HCl) were of analytical grade and purchased from Sinopharm Chemical Reagent Shanghai Co., Ltd. (Shanghai, China). Disodium hydrogen phosphate (Na_2_HPO_4_) and tris(hydroxymethyl) aminomethane (Tris) were of analytical grade and purchased from McLean Biochemical Technology Co., Ltd. (Shanghai, China). In-house-prepared, ultra-pure water was used for the experiments.

### 2.2. Preparation of PC@Mg-CaP

PC in Tris-HCl buffer (pH = 7.4) was co-incubated with 10 mM CaCl_2_ and 10 mM MgCl_2_ for 30 min. Then, 10 mM Na_2_HPO_4_ was slowly added to the solution according to the Ca/P ratio. Since the Ca/P ratio can affect the formation and stability of CaP, we selected values of 2, 3, and 5 for the preparation of PC@Mg-CaP. Tris-HCl buffer (pH = 7.4) was added to make up the volume. The mixture was stirred at 25 °C for 4 h to mineralize PC. The mineralized dispersion was centrifuged at 12,000 rpm at 4 °C for 10 min, and the supernatant was discarded. Subsequently, the precipitate was washed with ultra-pure water three times to obtain PC@Mg-CaP.

### 2.3. Characterization of PC@Mg-CaP

#### 2.3.1. Electron Microscopy Analysis 

PC solution and PC@Mg-CaP dispersion were added to the copper net with a carbon film. After drying at room temperature, the samples were negatively stained with phosphotungstic acid and dried under a tungsten lamp. Subsequently, transmission electron microscopy (TEM) analysis was carried out using a JEM-1200EX instrument (JEOL, Japan). The size of nanoparticles was obtained by statistical analysis of TEM images using Image-Pro Plus 6.0.

The PC@Mg-CaP with a Ca/P ratio = 5 and CaP were analyzed for surface elements using energy dispersive spectrometry (EDS). The samples did not require any staining for this analysis. EDS analyses were performed using an S-4800 instrument (Hitachi, Japan). 

#### 2.3.2. Laser Confocal Measurements

PC@Mg-CaP with Ca/P = 5 was placed in a special confocal dish for laser confocal microscopic measurements. Images were acquired using bright field and fluorescence channels. The excitation wavelength of the fluorescence channel was 561 nm and the magnification of the objective lens was 20×. Measurements were conducted using an A1R HD25 laser confocal microscope (Nikon, Japan).

#### 2.3.3. X-ray Diffraction Analysis

Freeze-dried PC@Mg-CaP with a Ca/P ratio = 5 was subjected to X-ray diffraction (XRD) analysis. The analytical parameters were as follows: scanning range: 2θ = 10–80°, scanning rate: 10°/min, voltage: 40 kV, and electric current: 10 mA.

#### 2.3.4. Fourier Transform Infrared (FTIR) Spectroscopy 

PC@Mg-CaP with different Ca/P ratios were frozen overnight in a freezer at −80 °C and was then vacuum freeze-dried for 24 h. PC@Mg-CaP and dry KBr were mixed and grounded. The spectra were recorded from a wavelength of 400 cm^−1^ to 4000 cm^−1^ for 64 runs. The spectra were recorded at a resolution of 4 cm^−1^. 

#### 2.3.5. Determination of Encapsulation Efficiency and Drug Loading

Encapsulation efficiency (EE) and loading capacity (LC) are important parameters of nanocarrier systems. EE was calculated based on the PC content in the solution before and after mineralization, using Equation (1) [14] as follows:

(1) $$\mathrm{EE}\;\left(\%\right)=\frac{m_{\mathrm{initial}}-{\;m}_{\mathrm{surplus}}}{m_{\mathrm{initial}}}\times 100\%$$

where m_initial_ is the initial PC content input and m_surplus_ is the PC content that is not mineralized in the supernatant after centrifugation. LC was calculated according to Equation (2) [29] as follows:

(2) $$\mathrm{LC}\;\left(\%\right)=\frac{m_{\mathrm{encapsulation}}}{m_{\mathrm{mineralized}\;\mathrm{microsphere}}}\times 100\%$$

where m_encapsulation_ is the PC mass in PC@Mg-CaP and the m_mineralized microsphere_ is the mass of PC@Mg-CaP after washing with ultra-pure water followed by centrifugation and drying.

#### 2.3.6. UV-Vis Spectroscopy

The PC concentration was determined by analyzing the UV-Vis spectra of the solution. The UV-Vis spectra were measured using a UV-2550 spectrophotometer (Shimadzu Instrument Co., Ltd., Suzhou, China). Since PC@Mg-CaP was dispersed in Tris-HCl (pH = 7.4) buffer, Tris-HCl buffer was used as the blank. The UV-Vis absorption spectra were measured at 450–750 nm. The PC concentration was calculated as shown in Equation (3) [2,14] as follows:

(3) $$C\;\left(\mathrm{mg}/\mathrm{mL}\right)=\frac{A_{620}-0.475\times A_{652}}{5.34}$$

where A_620_ and A_652_ are absorbance values of the solution at 620 nm and 652 nm, respectively. 

### 2.4. Thermal Stability Tests

#### 2.4.1. Heat Treatment 

To investigate the effect of temperature on the thermal stability of PC@Mg-CaP, the dispersions of PC@Mg-CaP with different Ca/P ratios were placed in a water bath at a constant temperature of 50 °C, 60 °C, or 70 °C. After heating for 30 min, they were immediately taken out and put in crushed ice to cool. After the samples were cooled to room temperature, the PC concentration of the sample was determined.

Furthermore, to explore the change in thermal stability of PC@Mg-CaP with the heating time, dispersions of PC@Mg-CaP with different Ca/P ratios were placed in a water bath at 60 °C. The heating time was 0–90 min, and samples were taken every 10 min. The samples were immediately placed on ice after taking them out of the water bath. When the sample temperature dropped to room temperature, the PC concentration of the sample was determined.

#### 2.4.2. Assessment of the Relative Concentration

The concentration calculations for PC were based on the excitation wavelength at 590 nm and the maximum emission wavelength between 600–750 nm. The slit width was fixed to 10 nm. Fluorescence spectra of PC@Mg-CaP before and after the heat treatment were acquired with a F-4600 Fluorescence spectrometer (Hitachi, Tokyo, Japan). The relative concentration (C_R_) was calculated, and the specific calculation method was shown in Equation (4) [30] as follows:

(4) $$C_{R}\;\left(\%\right)=\frac{C_{\mathrm{remaining}}}{C_{\mathrm{initial}}}\times 100\%$$

where C_remaining_ and C_initial_ are the concentrations of PC before and after the heat treatment, respectively. To evaluate the significance of thermal stability of samples with different treatments, all measurements were repeated three times, and the data were analyzed using the analysis of variance (ANOVA). 

### 2.5. Evaluation of Comprehensive Thermal Analysis 

The lyophilized sample (10–12 mg) was taken in a crucible. Differential calorimetric scanning (DSC) analysis was carried out in the temperature range of 20–250 °C, and the temperature was increased at 10 °C/min. The data were obtained using a DSC 200 PC (Netzsch Instruments, Selb, Germany). Thermogravimetric analysis (TGA) was used to measure the weight change in the sample at a heating rate of 10 °C/min until the sample reached a constant weight. TGA was conducted on a Q600 synchronous thermal analyzer (TA, New Castle, DE, USA).

### 2.6. Statistical Analysis

All experiments were performed based on more than three parallel replicates. Data were depicted using Origin 2021 (OriginLab Inc., Northampton, MA, USA). Statistical analyses of the experimental data were conducted using IBM SPSS Statistics 26 (IBM Inc., New York, NY, USA). Differences were analyzed with a two-way ANOVA and Tukey’s multiple comparison post-hoc test, using a significance test level of 5% (*p* < 0.05). 

## 3. Results 

### 3.1. PC@Mg-CaP Construction via the In Situ Mineralization of CaP

**Figure 1:**
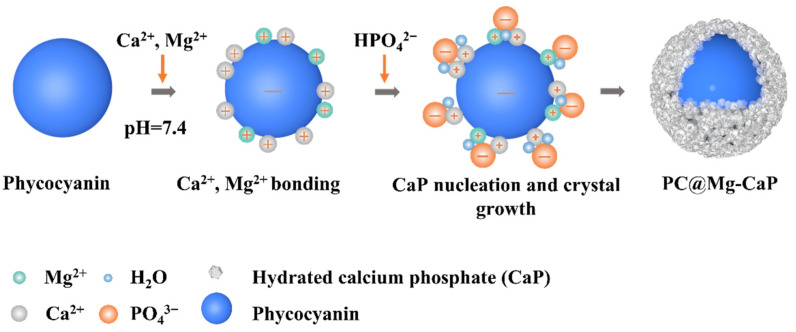
Schematic diagram of the method to construct PC@Mg-CaP by in situ mineralization of CaP.

Biological macromolecules with excessive negative charges are ideal templates for external mineralization [31]. Positively charged mineral ions can spontaneously be adsorbed onto the surface of the organisms through electrostatic interactions. Thus, nano mineral shells can be formed in situ on the surface of biomolecules [22,32,33]. PC is an acidic pigment-protein complex. Under physiological pH, PC carries abundant negative charges, which provides favorable conditions for the nucleation and crystallization of CaP. Moreover, the presence of glutamic acid, aspartic acid, and phosphorylated serine in PC might also be beneficial for CaP nucleation [34]. In this study, PC was used to induce the in situ mineralization of CaP on its surface to form a dense mineral shell (Figure 1).

### 3.2. Morphological Characterization of PC@Mg-CaP

**Figure 2:**
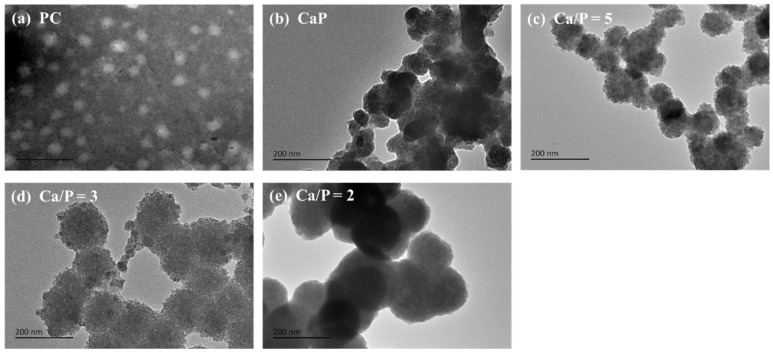
Electron microscopy characterization of PC@Mg-CaP. (**a**,**b**) TEM images of PC and CaP, respectively; (**c**–**e**) TEM images of PC@Mg-CaP with different Ca/P ratios, which were 5, 3, and 2, respectively.

To verify our hypothesis that it is feasible to prepare PC@Mg-CaP by in situ mineralization, we observed the morphology of mineralized PC by TEM. The Ca/P ratio could affect the quantity and formation of CaP crystals, thus influencing the size and shape of the nanoparticles [35]. Therefore, we selected Ca/P ratios of 2, 3, and 5 for experimental investigation with fixed Ca^2+^ content. PC was found to be ellipsoidal with a diameter of 45.8 ± 3.6 nm (Figure 2a), whereas CaP was unstable and existed as various shapes and sizes (Figure 2b). Notably, when PC was present, CaP spontaneously grew on the surface of the PC and formed a dense mineral coating. PC@Mg-CaP was spherical and prone to aggregation (Figure 2c–e). With a decrease in the Ca/P ratio, the diameter of mineralized PC increased continuously (Figure 2c–e). When the Ca/P was 5, 3, and 2, the diameters of PC@Mg-CaP were 107.9 ± 7.1 nm, 140.7 ± 12.6 nm, and 156.7 ± 15.5 nm, respectively. These results were related to the content of phosphate. As the phosphate content increased, the produced minerals accreted, leading to an increase in the thickness of the mineral shell [36]. In addition, during the formation of CaP, it will experience a variety of metastable phases. When the pH of the solution is around neutral, the initial phase state of CaP is amorphous calcium phosphate (ACP), and then it will change to more stable phases of CaP, such as octacalcium phosphate (OCP) and hydroxyapatite (HAP). Previous studies showed that ACP can better maintain the thermal stability of protein [26]. During the formation of ACP, Mg^2+^ can either adsorb onto, or incorporate into, the ACP precursor particles and significantly inhibit the transformation of ACP to other phases [37,38]. Therefore, the addition of Mg^2+^ could help to enhance the thermal stability of mineralized PC. These results of TEM confirmed the feasibility of preparing mineralized PC nanoparticles by in situ mineralization of CaP. The change in the size of the mineralized particles also indicated that the thickness of the CaP mineral shell could be regulated by changing the content of the phosphate.

### 3.3. Compositional Analysis of PC@Mg-CaP

**Figure 3:**
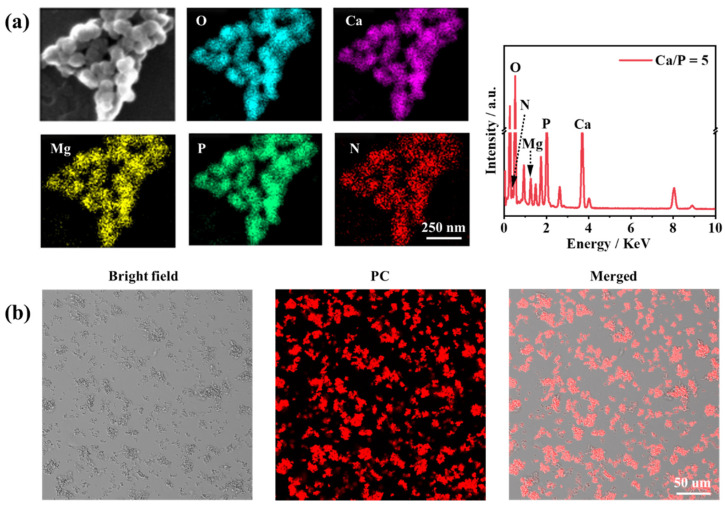
Composition analysis of PC@Mg-CaP. (**a**) Surface element mapping analysis of PC@Mg-CaP. (**b**) Laser confocal scanning microscopic images of PC@Mg-CaP. The fluorescence signal of PC was obtained with a 561 nm laser channel. The Ca/P ratio of the sample was 5.

The mineral shell of PC@Mg-CaP with a Ca/P ratio = 5 was thin and easy to analyze surface elements. To further confirm the composition of mineralized PC, the sample with a Ca/P ratio = 5 was selected to conduct surface element analysis and laser confocal microscopic measurements. We mainly analyzed five elements, including O, P, Mg, Ca, and N, during surface element analysis (Figure 3a, Table S1). Among them, N was the representative element of protein, whereas P, Ca, and Mg were the main elements in the CaP mineral. The results showed that all five elements were uniformly distributed on the surface of the nanoparticles. The high contents of Ca, Mg, and P confirmed the presence of CaP minerals on the surface of mineralized PC in abundant quantities. Notably, the presence of N was not observed in CaP particles (Figure S1, Table S2). However, N was detected in the mineralized PC. This proved that the mineralized nanoparticles were hybrid particles of protein and inorganic minerals. Because PC was wrapped in a thick and dense CaP shell, the content of N was low among the elements analyzed. Moreover, PC had fluorescent properties, which could be used to locate PC. The unique fluorescence emitted by the mineralized PC also verified our experimental synthesis of PC@Mg-CaP (Figure 3b). Moreover, the even distribution of Mg also demonstrated that Mg^2+^ participated in the formation of the mineral shell. The ratio of Ca/Mg was found to be approximately 2.47 (Table S1). The Ca/Mg ratio also affected the morphology, crystallinity, and thermal stability of minerals. With an increase in the Ca/Mg ratio, carbonate minerals tended to grow in a spherical shape, and both crystallinity and thermal stability increased [37]. Mg^2+^ was able to get adsorbed in or bind to ACP particles [38]. Furthermore, the carboxyl group of amino acids promoted the dehydration of Mg^2+^, thereby promoting the participation of Mg^2+^ in the formation of minerals.

### 3.4. Structural Analysis of PC@Mg-CaP

**Figure 4:**
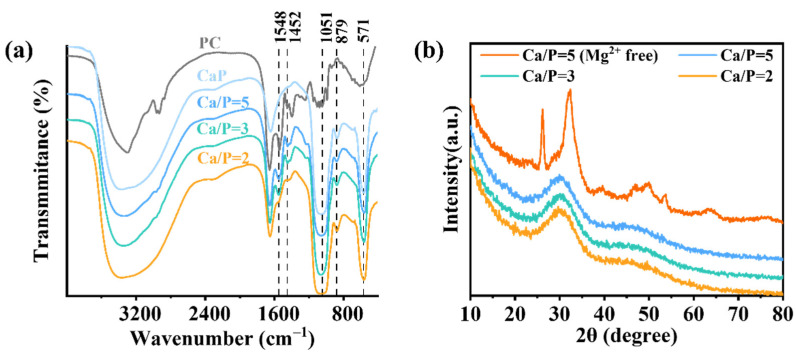
Structural characterizations of PC@Mg-CaP. (**a**) FTIR of PC, CaP, and PC@Mg-CaP with different Ca/P ratios. (**b**) XRD of PC@Mg-CaP with different Ca/P ratios. The Ca/P ratios were 5, 3, and 2, respectively.

To gain deeper insights into the structure of PC@Mg-CaP, FTIR and XRD analyses were performed (Figure 4). The characteristic peak for N-H bending vibration was observed at 1548 cm^−1^ [30]. The characteristic peak at 1452 cm^−1^ was attributed to C-N stretching vibration [39]. These were the characteristic peaks of PC. Moreover, the absorption peaks of CaP at 1051 cm^−^^1^ and 571 cm^−1^ were caused by PO_4_^3−^ stretching and bending vibrations, respectively [36,40]. The peak at 879 cm^−1^ was assigned to H-O(P) stretching in HPO_4_^2−^ ions [37,41]. As expected, PC@Mg-CaP showed FTIR characteristics of both PC and CaP (Figure 4a). With a decreasing Ca/P ratio, the characteristic peaks of PC in the mineralized protein became weaker. This might have stemmed from the thickening of the CaP shell on the surface of the mineralized PC. Further, the broadband at 2600–3600 cm^−1^ was ascribed to O-H stretching in water molecules [37]. Interestingly, compared with that of PC, PC@Mg-CaP had a broader band at 2600–3600 cm^−1^, which suggested that PC@Mg-CaP might contain more water. This might be due to the high amount of water molecules trapped when CaP shells were formed [24]. Considering the existence of CaP polycrystalline phase, we analyzed the crystal structure of the PC@Mg-CaP by XRD (Figure 4b). The peaks at 2θ = 26° and 32° in XRD spectrum of PC@CaP with Ca/P = 5 indicated that the formed CaP shell of the mineralized PC without Mg^2+^ was HAP. However, when the mineralized solution contained Mg^2+^, the samples of the mineralized PC with different Ca/P ratios had a common wide peak at 2θ = 30° indicating that the CaP shell of the mineralized PC existed as ACP (Ca_x_H_y_(PO_4_)_z_ · nH_2_O). The difference between the samples was closely related to the phase transition of CaP. When the pH of the solution was approximately neutral, excessive Ca^2+^ and PO_4_^3−^ would form minerals with ACP as the initial phase [35]. Importantly, Mg^2+^ could inhibit the phase transition of CaP, acting as a stabilizer [38]. In summary, these results indicated the shell of PC@Mg-CaP was ACP, and the existence of Mg^2+^ was crucial to maintain ACP.

### 3.5. Evaluation of the Efficacy of Mineralization

To evaluate the ability and efficacy of PC to induce the in situ mineralization of CaP, the EE and LC of the mineralized PC were determined. Table 1 shows the EE and LC of PC@Mg-CaP with different Ca/P ratios. With a decrease in the Ca/P ratio, the EE of PC@Mg-CaP increased, but LC decreased. When the Ca/P ratio was 5, the EE and LC of PC@Mg-CaP were 59.19% and 11.86%, respectively, whereas for the Ca/P ratio of 2, the EE increased by 13.39% and LC decreased by 5.09%. With an increase in phosphate radicals, the amount of insoluble phosphate that precipitated increased. Subsequently, more PC was encapsulated, resulting in an increase in the EE. In addition, the CaP shell on the protein surface was thickened; this led to a reduction in the LC of PC@Mg-CaP. Moreover, when the Ca/P ratio was less than 3, the EE was greater than 70%, indicating that PC induced CaP in situ mineralization is a highly efficient coating method.

### 3.6. Enhancement of the Thermal Stability of PC by the Mineralization of CaP

**Figure 5:**
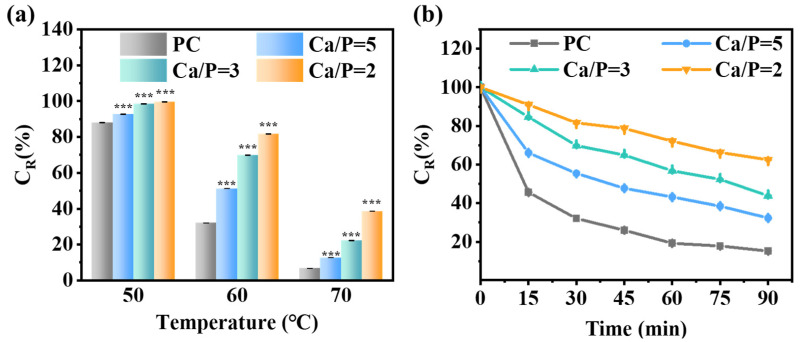
Characterization of PC and PC@Mg-CaP thermal stability. (**a**) C_R_ changes in PC and PC@Mg-CaP (Ca/P = 5, 3, 2) after being heated at 50 °C, 60 °C, and 70 °C for 30 min, respectively. (**b**) C_R_ changes in PC and PC@Mg-CaP (Ca/P = 5, 3, 2) overtime at 60 °C. The representative data shown were the mean ± SD, and PC was set as the control. Differences in C_R_ were analyzed with a two-way ANOVA and Tukey’s multiple comparison post-hoc test. *** *p* < 0.001.

Inadequate thermal stability has been a considerable barrier in the expansion of PC applications. To analyze the effect of mineralization on the thermal stability of PC, we measured the changes in C_R_ of PC and PC@Mg-CaP under different heating conditions (Figure 5). Figure 5a showed the effect of heating temperature on the thermal stability of mineralized PC. With an increase in the heating temperature, the C_R_ of PC and PC@Mg-CaP decreased. Importantly, the C_R_ of PC@Mg-CaP was consistently significantly higher than that of PC (*p* < 0.05), and the thermal stability of PC@Mg-CaP was better at a lower Ca/P ratio. When heating at 70 °C for 30 min, the C_R_ of PC was only 6.76%. However, CaP mineralization was able to preserve the PC activity. The C_R_ of PC@Mg-CaP with a Ca/P ratio of 5, 3, and 2 was 1.88, 3.28, and 5.31 times that of PC, respectively (Figure 5a). In addition, we also studied the C_R_ changes of mineralized PC overtime at 60 °C. Figure 5b showed that with the increase of heating time, C_R_ of PC and PC@Mg-CaP all decreased, but the C_R_ descent rate of PC was faster than that of PC@Mg-CaP. The C_R_ of PC was only 45.61% after heating at 60 °C for 15 min. However, when heated at 60 °C for 90 min, the C_R_ of PC@Mg-CaP with a Ca/P ratio of 2 was still 62.49% (Figure 5b). These results suggested that mineralization could greatly improve the thermal stability of PC, and that the Ca/P ratio was the key to regulating the thermal stability of mineralized PC. These experimental results proved that CaP in situ mineralization was an effective approach to enhance the thermal stability of PC.

### 3.7. Enhancement of the Fluorescence Stability of PC by the Mineralization of CaP 

**Figure 6:**
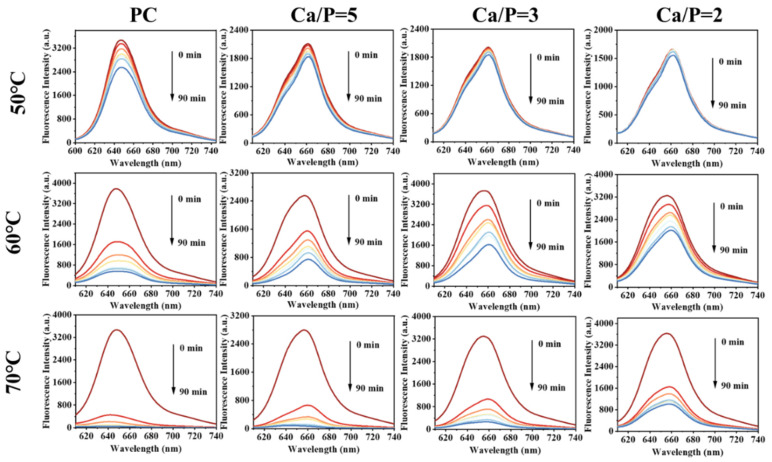
Fluorescence spectra changes of PC and PC@Mg-CaP (Ca/P = 5, 3, 2) with time at different heating temperatures. The heating temperature was 50 °C, 60 °C, and 70 °C, respectively. The heating time was 0–90 min. The fluorescence spectrum was recorded every 10 min. The excitation wavelength was set to 590 nm, and the slit width was fixed to 10 nm.

Heat can lead to a reduction in PC fluorescence, which decreases the efficiency of PC as a fluorescent probe. We investigated the effect of mineralization on the stability of PC with heating (Figure 6). PC showed low fluorescence stability when the temperature reached 50 °C. With an increase in the temperature, the fluorescence characteristics were gradually lost during heating. The fluorescence stability of the mineralized PC was higher at 50 °C, with that of PC@Mg-CaP with a Ca/P ratio = 2 being the highest. When heated at 50 °C for 90 min, the fluorescence intensity of PC decreased by 26.66%, but that of PC@Mg-CaP with a Ca/P ratio = 2 only decreased by 6.22%. When the temperature was higher than 50 °C, the fluorescence stability of PC@Mg-CaP declined rapidly. When the heating temperature was 60 °C, the fluorescence intensity of PC was reduced by 54.69%, and that of PC@Mg-CaP with a Ca/P ratio = 2 was reduced by 9.45% upon heating for 15 min. These results proved that the in situ mineralization of CaP could enhance the fluorescence stability of PC. Notably, we observed that the fluorescence characteristic peak of PC was red-shifted after mineralization. This indicated that the hydrophobicity of the PC increased after mineralization. Moreover, the CD spectra of PC@Mg-CaP with different Ca/P ratios showed similar trends compared with that of PC. However, PC@Mg-CaP samples underwent a loss of α-helix structure as indicated by the reduced peak intensity at 208 nm and 222 nm after mineralization (Figure S2). These might have been because the hydrophobic part of the protein was more conducive to CaP adsorption [42]. Mineralization promoted the extension and exposure of hydrophobic groups of PC, made them easy to interact with an aqueous medium, and led to conformational changes of proteins [43].

### 3.8. Analysis of the Thermal Properties of PC@Mg-CaP

**Figure 7:**
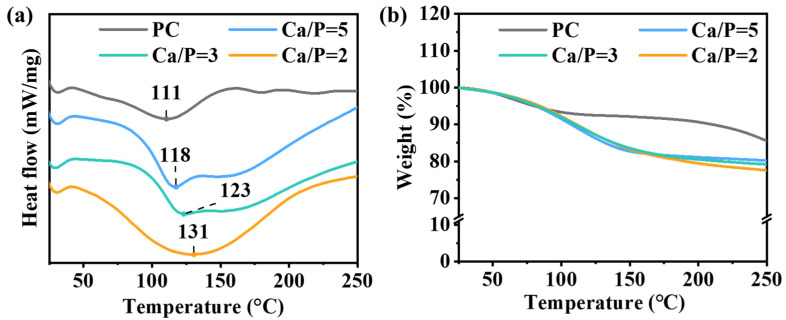
Thermal characterization of PC and PC@Mg-CaP. DSC (**a**) and TGA (**b**) data of PC and PC@Mg-CaP. The ratios of Ca/P were 5, 3, and 2, respectively. The temperature was raised at 10 °C/min.

To clarify the mechanism underlying CaP mineralization-mediated improvements in PC thermal stability, we explored the thermal properties of PC@Mg-CaP by TGA and DSC. The denaturation temperature of PC was 111 °C. After CaP mineralization, the denaturation temperature increased by 7–20 °C (Figure 7a). The results showed that mineralization could effectively improve the thermal stability of PC. With an increasing temperature, the weight of PC and PC@Mg-CaP quickly decreased (Figure 7b). When the temperature reached 110 °C, the weight loss of PC was approximately 7.2%, and that of PC@Mg-CaP was about 10%. When the temperature continued to rise, the weight loss of PC slowed down; however, the weight of PC@Mg-CaP continued to decrease rapidly, until the temperature reached approximately 180 °C. At this point, the weight loss of PC@Mg-CaP with different Ca/P ratios was between 18.5–19.3%; the descending rate slowed down subsequently. It is worth noting that these results indicate that the water molecules were more abundant in PC@Mg-CaP and were more difficult to remove. This could be attributed to the ACP shell of PC@Mg-CaP containing more water. Some of the water existed in the gaps between CaP clusters, and the remaining water molecules were adsorbed on the surface of CaP [44]. These restricted water molecules were more stable than free water molecules in the environment. Compared with PC, PC@Mg-CaP showed more significant endothermic peaks during heating, which suggested that the water molecules needed to absorb extensive heat to be liberated (Figure 7a). Previous reports have also indicated that reductive polysaccharides such as trehalose could improve the thermal stability of proteins and other biological macromolecules by confining the fluidity of water molecules [45]. Consequently, the relatively stable microenvironment provided by the CaP shell was the main reason underlying the in situ mineralization-mediated enhancement in PC thermal stability.

## 4. Conclusions

In this study, we successfully prepared Mg-doped CaP-coated PC nanoparticles by the in situ mineralization. We also characterized the structure of mineralized PC and evaluated the effects of mineralization on the structure and thermal stability of PC. The study indicated that the PC@Mg-CaP nanoparticles had a core-shell structure. The dense shell was composed of ACP and was regulated by Mg^2+^ during the formation. The mineralization of CaP caused a loss of α-helix structure and an increase in hydrophobicity of PC. Mineralization could significantly improve the thermal stability of PC. Notably, the Ca/P ratio was critical to regulate the thermal stability of PC@Mg-CaP. The thermal stability of PC@Mg-CaP with varied Ca/P ratios in the following sequence was: Ca/P = 2 > Ca/P = 3 > Ca/P = 5. Furthermore, the results showed that the dense CaP shell, which contained abundant bound water, also provided a relatively closed microenvironment for PC. It could reduce the damage of external heat to the structure of PC. That may be the underlying mechanism for the enhancement of the thermal stability of mineralized PC. This study proved that the in situ mineralization of CaP is a safe, facile, and effective method to improve the thermal stability of PC. It also provides a feasible mineralization-based strategy to improve the thermal stability of thermosensitive material.

## Figures and Tables

**Table 1:** EE and LC of PC@Mg-CaP with different Ca/P ratios.

Index	Ca/P = 5	Ca/P = 3	Ca/P = 2
EE (%)	59.19 ± 0.55	72.25 ± 0.31	72.58 ± 0.07
LC (%)	11.86 ± 2.00	8.51 ± 1.20	6.77 ± 0.35

## Data Availability

The datasets used and/or analyzed during the current study are available from the corresponding author on reasonable request.

## Supplementary Materials

The following are available online at https://www.mdpi.com/article/10.3390/foods11040503/s1, Figure S1: Surface element mapping (a–e) and energy spectrum analysis (f) of CaP, Figure S2. UV-circular dichroism spectrum of PC and PC@Mg-CaP, Table S1: Elements proportion of PC@Mg-CaP (Ca/P = 5) sample surface, Table S2: Elements proportion of CaP sample surface.

## References

[1] Wu X.J., Yang H., Chen Y.T., Li P.P. (2018). Biosynthesis of fluorescent beta subunits of C-phycocyanin from spirulina subsalsa in escherichia coli, and their antioxidant properties. Molecules.

[2] Wu H.L., Wang G.H., Xiang W.Z., Li T., He H. (2016). Stability and antioxidant activity of food-grade phycocyanin isolated from spirulina platensis. Int. J. Food Prop..

[3] Romay C., González R., Ledón N., Remirez D., Rimbau V. (2003). C-Phycocyanin: A biliprotein with antioxidant, anti-inflammatory and neuroprotective effects. Curr. Protein Pept. Sci..

[4] Fernandes E.S.E., Figueira F.D.S., Lettnin A.P., Carrett-Dias M., Filgueira D., Kalil S., Trindade G.S., Votto A.P.S. (2017). C-Phycocyanin: Cellular targets, mechanisms of action and multi drug resistance in cancer. Pharmacol. Rep..

[5] Ma P., Huang R., Jiang J., Ding Y., Li T., Ou Y. (2020). Potential use of C-phycocyanin in non-alcoholic fatty liver disease. Biochem. Biophys. Res. Commun..

[6] Jespersen L., Strømdahl L.D., Olsen K., Skibsted L.H. (2005). Heat and light stability of three natural blue colorants. Eur. Food Res. Technol..

[7] Sozer N., Kokini J.L. (2009). Nanotechnology and its applications in the food sector. Trends Biotechnol..

[8] Pradeep H.N., Nayak C.A. (2019). Enhanced stability of C-phycocyanin colorant by extrusion encapsulation. J. Food Sci. Technol..

[9] Manconia M., Pendas J., Ledon N., Moreira T., Sinico C., Saso L., Fadda A.M. (2009). Phycocyanin liposomes for topical anti-inflammatory activity: In-vitro in-vivo studies. J. Pharm. Pharmacol..

[10] Purnamayati L., Dewi E.N., Kurniasih R.A. (2018). Phycocyanin stability in microcapsules processed by spray drying method using different inlet temperature. IOP Conf..

[11] Schmatz D.A., Mastrantonio D.J.D.S., Costa J.A.V., Morais M.G.D. (2020). Encapsulation of phycocyanin by electrospraying: A promising approach for the protection of sensitive compounds. Food Bioprod. Process..

[12] Sercombe L., Veerati T., Moheimani F., Wu S.Y., Sood A.K., Hua S. (2015). Advances and challenges of liposome assisted drug delivery. Front. Pharmacol..

[13] Manconi M., Mura S., Manca M.L., Fadda A.M., Dolz M., Hernandez M.J., Casanovas A., Diez-Sales O. (2010). Chitosomes as drug delivery systems for C-phycocyanin: Preparation and characterization. Int. J. Pharm..

[14] Lemos P.V.F., Opretzka L.C.F., Almeida L.S., Cardoso L.G., Silva J., Souza C.O., Villarreal C.F., Druzian J.I. (2020). Preparation and characterization of C-phycocyanin coated with STMP/STPP cross-linked starches from different botanical sources. Int. J. Biol. Macromol..

[15] Marie B., Joubert C., Tayale A., Zanella-Cleon I., Belliard C., Piquemal D., Cochennec-Laureau N., Marin F., Gueguen Y., Montagnani C. (2012). Different secretory repertoires control the biomineralization processes of prism and nacre deposition of the pearl oyster shell. Proc. Natl. Acad. Sci. USA.

[16] Tambutté S., Holcomb M., Ferrier-Pagès C., Reynaud S., Tambutté É., Zoccola D., Allemand D. (2011). Coral biomineralization: From the gene to the environment. J. Exp. Mar. Biol. Ecol..

[17] Liu Z., Xu X., Tang R. (2016). Improvement of biological organisms using functional material shells. Adv. Funct. Mater..

[18] Li B., Cui Y., Wang X., Tang R. (2021). Novel nanomaterial-organism hybrids with biomedical potential. WIREs Nanomed. Nanobiotechnol..

[19] Nudelman F., Sommerdijk N.A.J.M. (2012). Biomineralization as an inspiration for materials chemistry. Angew. Chem. Int. Ed..

[20] Hu D., Ren Q., Li Z., Zhang L. (2020). Chitosan-based biomimetically mineralized composite materials in human hard tissue repair. Molecules.

[21] Sokolova V., Epple M. (2021). Biological and medical applications of calcium phosphate nanoparticles. Chem. Eur. J..

[22] Wang G., Cao R.Y., Chen R., Mo L., Han J.F., Wang X., Xu X., Jiang T., Deng Y.Q., Lyu K. (2013). Rational design of thermostable vaccines by engineered peptide-induced virus self-biomineralization under physiological conditions. Proc. Natl. Acad. Sci. USA.

[23] Youn W., Kim J.Y., Park J., Kim N., Choi H., Cho H., Choi I.S. (2020). Single-cell nanoencapsulation: From passive to active shells. Adv. Mater..

[24] Habraken W., Habibovic P., Epple M., Bohner M. (2016). Calcium phosphates in biomedical applications: Materials for the future?. Mater. Today.

[25] Salama A., El-Sakhawy M. (2014). Preparation of polyelectrolyte/calcium phosphate hybrids for drug delivery application. Carbohydr. Polym..

[26] Yang Y., Wang G., Zhu G., Xu X., Pan H., Tang R. (2015). The effect of amorphous calcium phosphate on protein protection against thermal denaturation. Chem. Commun..

[27] Wang G., Wang H.J., Zhou H., Nian Q.G., Song Z., Deng Y.Q., Wang X., Zhu S.Y., Li X.F., Qin C.F. (2015). Hydrated silica exterior produced by biomimetic silicification confers viral vaccine heat-resistance. ACS Nano.

[28] Cai A.Y., Zhu Y.J., Qi C. (2020). Biodegradable Inorganic Nanostructured Biomaterials for Drug Delivery. Adv. Mater. Interfaces.

[29] Hadiyanto H., Christwardana M., Suzery M., Sutanto H., Nilamsari A.M., Yunanda A. (2019). Effects of carrageenan and chitosan as coating materials on the thermal degradation of microencapsulated phycocyanin from *Spirulina* sp. Int. J. Food Eng..

[30] Tong X., Prasanna G., Zhang N., Jing P. (2020). Spectroscopic and molecular docking studies on the interaction of phycocyanobilin with peptide moieties of C-phycocyanin. Spectrochim. Acta Part A Mol. Biomol. Spectrosc..

[31] Gal A., Wirth R., Barkay Z., Eliaz N., Scheffel A., Faivre D. (2017). Templated and self-limiting calcite formation directed by coccolith organic macromolecules. Chem. Commun..

[32] Knuschke T., Bayer W., Rotan O., Sokolova V., Wadwa M., Kirschning C.J., Hansen W., Dittmer U., Epple M., Buer J. (2014). Prophylactic and therapeutic vaccination with a nanoparticle-based peptide vaccine induces efficient protective immunity during acute and chronic retroviral infection. Nanomedicine.

[33] Liang K., Richardson J.J., Cui J., Caruso F., Doonan C.J., Falcaro P. (2016). Metal-organic framework coatings as cytoprotective exoskeletons for living cells. Adv. Mater..

[34] Chu X., Jiang W., Zhang Z., Yan Y., Pan H., Xu X., Tang R. (2011). Unique roles of acidic amino acids in phase transformation of calcium phosphates. J. Phys. Chem. B.

[35] Sugiura Y., Onuma K., Kimura Y., Miura H., Tsukamoto K. (2011). Morphological evolution of precipitates during transformation of amorphous calcium phosphate into octacalcium phosphate in relation to role of intermediate phase. J. Cryst. Growth.

[36] Wang X., Sun C., Li P., Wu T., Zhou H., Yang D., Liu Y., Ma X., Song Z., Nian Q. (2016). Vaccine engineering with dual-functional mineral shell: A promising strategy to overcome preexisting immunity. Adv. Mater..

[37] Gelli R., Briccolani-Bandini L., Pagliai M., Cardini G., Ridi F., Baglioni P. (2021). Exploring the effect of Mg^2+^ substitution on amorphous calcium phosphate nanoparticles. J. Colloid Interface Sci..

[38] Ding H., Pan H., Xu X., Tang R. (2014). Toward a detailed understanding of magnesium ions on hydroxyapatite crystallization inhibition. Cryst. Growth Des..

[39] Wen Y., Wen P., Hu T.G., Linhardt R.J., Zong M.H., Wu H., Chen Z.Y. (2020). Encapsulation of phycocyanin by prebiotics and polysaccharides-based electrospun fibers and improved colon cancer prevention effects. Int. J. Biol. Macromol..

[40] Yuan X., Zhu B., Tong G., Su Y., Zhu X. (2013). Wet-chemical synthesis of Mg-doped hydroxyapatite nanoparticles by step reaction and ion exchange processes. J. Phys. Chem. B.

[41] Combes C., Rey C. (2010). Amorphous calcium phosphates: Synthesis, properties and uses in biomaterials. Acta Biomater..

[42] Wertz C.F., Santore M.M. (2001). Effect of surface hydrophobicity on adsorption and relaxation kinetics of albumin and fibrinogen: Single-species and competitive behavior. Langmuir.

[43] Norde W., Giacomelli C.E. (2000). BSA structural changes during homomolecular exchange between the adsorbed and the dissolved states. J. Biotechnol..

[44] Du L.W., Bian S., Gou B.D., Jiang Y., Huang J., Gao Y.X., Zhao Y.D., Wen W., Zhang T.L., Wang K. (2013). Structure of clusters and formation of amorphous calcium phosphate and hydroxyapatite: From the perspective of coordination chemistry. Cryst. Growth Des..

[45] Faieta M., Neri L., Sacchetti G., Di Michele A., Pittia P. (2020). Role of saccharides on thermal stability of phycocyanin in aqueous solutions. Food Res. Int..

