# Supplementary material for: Preparation and Characterization of Mg-Doped Calcium Phosphate-Coated Phycocyanin Nanoparticles for Improving the Thermal Stability of Phycocyanin
Source: Foods. 2022 Feb 10;11(4):503. doi: 10.3390/foods11040503 (PMC8871242; doi:10.3390/foods11040503)
Supplement: Supplementary file 1 [file foods-11-00503-s001.zip › foods-1544378-supplementary.pdf]

Supplementary Information

Preparation and Characterization of Mg-Doped Calcium Phosphate-Coated Phycocyanin Nanoparticles for Improving the Thermal Stability Of Phycocyanin

Qian Li [1], Ping Dong [1*], Laihao Li [2*]

[1] College of Food Science and Engineering, Ocean University of China, Qingdao 266003, China

[2] Key Laboratory of Aquatic Product Processing, Ministry of Agriculture and Rural Affairs, National R&D Center for Aquatic Product Processing, South China Sea Fisheries Research Institute, Chinese Academy of Fishery Sciences, Guangzhou 510300, China

Table S1. Elements proportion of PC@Mg-CaP (Ca/P = 5) sample surface

Element	Weight%	Weight% σ	Atomic %
N	6.98	0.61	8.98
O	69.95	0.47	78.72
Mg	2.92	0.05	2.16
P	8.28	0.08	4.81
Ca	11.87	0.11	5.33
Total	100.00		100.00

Table S2. Elements proportion of CaP sample surface

Element	Weight%	Weight% σ	Atomic %
O	83.68	0.21	91.64
Mg	1.59	0.02	1.15
P	6.03	0.03	3.41
Ca	8.70	0.04	3.80
Total	100.00		100.00

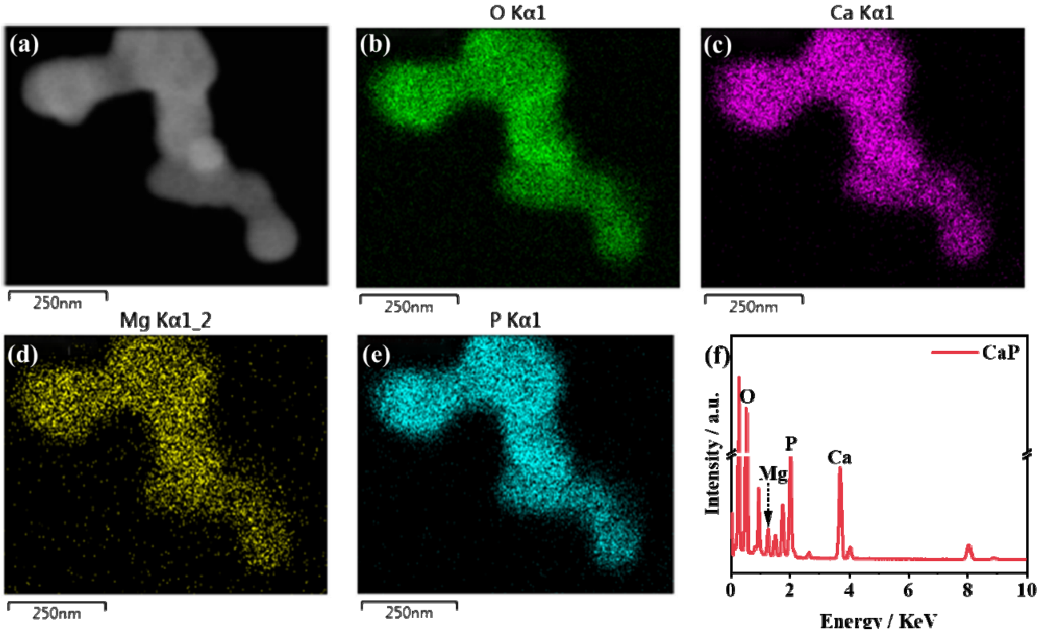

Figure S1. Surface element mapping (a-e) and energy spectrum analysis (f) of CaP.

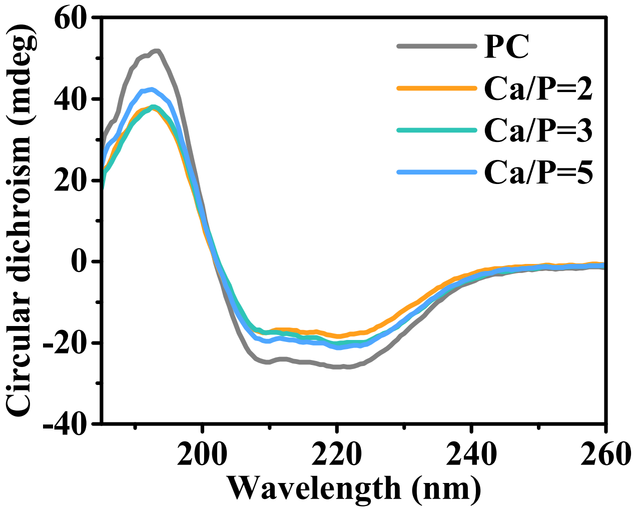

Figure S2. UV-circular dichroism spectrum of PC and PC@Mg-CaP.
